# Treatment of Neuralgia by the Valerianate of Ammonia

**Published:** 1856-10

**Authors:** 


### *Treatment of Neuralgia by the Valerianate of Ammonia.*

(Translated for the Examiner.)

Dr. Declat relates, in the Bulletin de Therapeutique, several cases which prove the happy effects that may be anticipated from the use of the above remedy.

CASE 1st. Madame the Marchioness of Fontanelle, (the lady has authorised us to give her name,) was attacked six years ago with facial neuralgia of the most severe description. The pain was first experienced while cutting a wisdom tooth, which was late in making its appearance. As this tooth came through in a wrong direction, Drs. A. Legrand and Jobert de Lamballe ordered its extraction. The pain was so violent that Madame de Fontanelle was unable to open her mouth, and they were obliged to give her chloroform.

In presence of the consulting physicians, Mr. Evans performed the operation of extraction with great skill. After the removal of the tooth, the neuralgia still continued. All the ordinary means were employed to relieve it; internally sulphate of quinine, opium, belladona, sulphate of strychnia, iron, gold, and quin-quina were administered, as well as external applications of opium plaster, blisters, morphia, dulcamara, chloroform, collodion, aconite, &c. &c.

Professors Sedillot and Velpeau saw the patient without being able to give her any relief. Professor Jobert de Lamballe proposed and obtained permission to apply the actual cautery over the course of the inferior maxillary nerve. This treatment, so terrifying to a woman, slightly lessened the acuteness of the pain, but failed to overcome it; and though suffering less, Madame de Fontanelle could neither eat nor sleep; being compelled, during at least six months, to have recourse to nutritive enemata, and tonic baths to preserve her health and life.

The waters of Plombiéres diminished, for a time, the frequency of the pains; during the second season, no benefit was derived from their action; the third, her malady was increased. She was in this suffering state when, on the 19th December, 1855, she was placed under my care.

The amelioration of her complaint produced by the waters of

Plombiéres during the first season, determined me to use Fowler's solution.

The invalid consented the more willingly to this means as she preferred death, she said, to insanity from suffering. One of her friends, also, Madame de Balzac, had written to her from Germany, that this remedy was in frequent use in the country in which she lived, and that it had, to her knowledge, cured more than one case of neuralgia.

From the 19th to the 22d of December, 12 drops in the morning, 12 drops at noon, and 12 drops in the evening of the following mixture were given her:

| | |
|---|---|
| Fowler's solution, - - - - | $\frac{1}{4}$ |
| Mint water, - - - - - | $\frac{3}{4}$ |

On the 22d, there was a little improvement, but the tongue was red, and she suffered much pain in the stomach; Madame de Fontanelle would not consent to diminish the next dose, as the slight amelioration she had experienced made her sanguine of more complete relief.

On the 24th, vomiting, diarrhœa, cramps in the stomach and pains returned. We discontinued the medicine.

On the 3d of January, 1856, the agony was unendurable, and my patient was in despair. I tried the valerianate of ammonia.

A tea-spoonful, taken in the evening, modified the pain at night, and rendered it bearable. Two tea-spoonsful the next day gave complete relief.

On the 6th of January, the patient could rise and speak.

On the 19th, she half opened her mouth and began to eat. The 3d of February, Madame de Fontanelle came up to me smilingly, and welcomed me, saying, "Dr. I have been well enough to dine in town; I can laugh; in society they look upon me as one risen from the dead." We gradually increased the dose to a dessert spoonful morning and evening; the improvement now became so great that her countenance resumed an entirely different aspect, and her appetite came back as hope returned.

Finally, on the 6th of May, the pains having for several days entirely ceased, we suspended the use of the medicine. Several weeks passed without a single twinge; but at the return of the first pain, the Marchioness snatched the bottle and took a dose of the valerianate.

From time to time, some shooting pains were felt; but every time the valerianate was resorted to they disappeared. The improvement continues, and there is nothing to cause us to anticipate that the remedy will lose its efficacy should the disease return.

The case given above is one of importance. From the first the patient had been attended with care, and even affection, by the most eminent physicians; for six years almost every known means had been employed, without results of any duration.

This case of neuralgia was much more obstinate and unmanageable from its being an hereditary affection. Madame de Fontanelle's mother had suffered fearfully from a similar neuralgia. Her brother, the Count of Essex, (sic !) has had tic doloureux from his youth ; and he is as well known in England from the great suffering he has gone through from it as from his high position.

Doctor A. Legrand has, throughout, watched this cure which he had pronounced hopeless six years ago ; wishing himself to verify the value of the new medicine, he ordered it in the same doses to Madame de V——, whom he considered equally incurable. We know that the relief has been quite prompt ; but we understand, from his having too rapidly increased the dose, that some cerebral disturbance was produced. These symptoms, however, disappeared as soon as the valerianate was given in proper doses. At present, Madame de V—— considers herself cured.

Case II. M. E. Letellier accompanied his wife to Plombières. During his sojourn at the waters, in the beginning of October, he was attacked with a pain in the head ; this pain extended to the muscles of the neck, passed through the top of the head and lost itself in the branches of the facial nerve. It was impossible for the patient to raise his head from the pillow. Various remedies were tried at Plombiéres, but the pains increased and the sufferer was taken back to Paris.

The least movement was so painful to him that he could only bear the journey by having his head supported by Madame Letellier's hands.

Dr. Louis tried blisters, sage, quinine and morphia, without any effect. To relieve his pain, M. Letellier took so much morphia as to fall into an alarming state of torpor.

On the first of October, 1855, we found the sick man in a state of extreme agony; he had not taken any morphia for two days, and suffered constantly.

On the same day, we began to use the valerianate of ammonia, two tea-spoonsful during the day in a half glass of eau sucrée. That night he had a little rest.

By continuing the use of the valerianate without increasing the dose, the patient was able to get up at the end of five days. On the ninth day he walked out to take a bath; he no longer felt any pain except in his neck and the back of his head; his nights became comfortable, his abilities and aptitude for business entirely restored.

Finally, from the 24th of the same month we discontinued our visits.

We met him again recently, and he tells us that he has had some slight twinges, which are at once dissipated by a spoonful of the valerianate.—*La Revue Médicale Francaise et Etrangére.*

